# Antisera-Neutralizing Capacity of a Highly Evolved Type 2 Vaccine-Derived Poliovirus from an Immunodeficient Patient

**DOI:** 10.3390/v16111761

**Published:** 2024-11-12

**Authors:** Yanan Wu, Runfang Zhang, Guangbo Yuan, Lingyu He, Xiaohu Dai, Hongyun Chuan, Mingqing Wang, Jing Liu, Lilan Xu, Guoyang Liao, Weidong Li, Jian Zhou

**Affiliations:** 1 Key Laboratory of Vaccine Research and Development for Major Infectious Diseases in Yunnan Province, Institute of Medical Biology, Chinese Academy of Medical Sciences, Peking Union Medical College, Kunming 650000, China; wuyanancx88@126.com (Y.W.); zrf_0625@163.com (R.Z.); m18286354721@163.com (G.Y.); daixiaohu@imbcams.com.cn (X.D.); liujing@imbcams.com.cn (J.L.); xulilan0624@imbcams.com.cn (L.X.); liaogy@imbcams.com.cn (G.L.); 2 Institute of Medical Biology, Kunming Medical University, Kunming 650500, China; 3 Department of Industrial Transformation, Institute of Medical Biology, Chinese Academy of Medical Sciences, Peking Union Medical College, Kunming 650000, China; 17787101074@163.com; 4 Production Department, Institute of Medical Biology, Chinese Academy of Medical Sciences, Peking Union Medical College, Kunming 650000, Chinalwd@imbcams.com.cn (W.L.)

**Keywords:** poliovirus, type II, pseudovirus, neutralizing antibodies, immunogenicity

## Abstract

Background: The serotype 2 oral poliovirus vaccine (OPV2) can revert to regain wild-type neurovirulence and spread, causing the emergence of vaccine-derived poliovirus (VDPV2) and immunodeficiency-related vaccine-derived polioviruses (iVDPVs). In the United States, testing carried out by the CDC of type II iVDPV (iVDPV2) with human immune serum from the vaccine has shown that the presence of the virus poses a threat to eradication efforts. Methods: We analyzed the major neutralization sites of VP1, VP2, and VP3 of the iVDPV using bioinformatics techniques and homology modeling (SWISS-MODEL). The three amino acid residues 679, 680, and 141 of the P1 region changed, which had an impact on the spatial conformation of the viral-neutralizing site. We tested polio-vaccinated human sera and rabbit anti-Sabin II polyantibodies against a panel of iVDPV pseudoviruses. Results: The results demonstrated that the serum’s capacity to neutralize mutant pseudoviruses diminished when amino acid substitutions were introduced into the P1 encapsidated protein, particularly when 141 and 679 were mutated together. This study emphasizes the significance of continually monitoring individuals who are known to be immunocompromised and maintaining high vaccination rates in OPV-using communities.

## 1. Introduction

Poliovirus (PV) is the cause of poliomyelitis, which destroys motor neurons in the central nervous system, causing paralysis and even death. Since 1954, the live oral PV vaccine (OPV) or the inactivated PV vaccine (IPV) from the Salk strain (wIPV) and Sabin strain (sIPV) have been effective vaccines for the prevention of polio [1]. In 1988, the World Health Organization launched its initiative to eradicate polio. By 2024, only two neighboring countries (Afghanistan and Pakistan) will need to interrupt the transmission of wild poliovirus type I [2]. The genetic instability of the OPV means that it can regain neurovirulence by reversing mutations, causing vaccine-associated paralytic poliomyelitis in vaccine recipients or their contacts. However, the incidence of vaccine-associated poliomyelitis is low [3]. These attenuated strains can also undergo genetic recombination during replication with other polio or non-polio human enteroviruses and evolve into vaccine-derived polioviruses (VDPVs) with a neurovirulent phenotype similar to that of wild poliovirus (WPV) [4]. IPV induces effective humoral immunity that protects against disease but does not always prevent subsequent encounters with WPV, allowing continued transmission in the population [5].

The ability of the OPV to induce mucosal immunity, due to its low cost and convenient administration, has made it the main weapon in the fight against poliovirus [6]. However, the OPV can mutate into strains that regain neurovirulence, posing a challenge to global polio eradication [7,8]. Many polio outbreaks associated with circulating vaccine-derived polioviruses (cVDPVs) have been observed in areas with low vaccine coverage [9].

In order to determine whether sIPV could block the infection by prevalent wild strains, Liao et al. commissioned the US CDC to conduct a wild strain cross-neutralization test using 18 strains and the Chinese CDC to conduct a cross-neutralization test with the wild strain XJ2011 imported from Pakistan [10]. sIPV produced an anti-polio serum with a neutralizing antibody potency well above the protective level (neutralizing antibody titer ≥ 1:8). The results shown in the research demonstrate that in the neutralization assay of a type II iVDPV strain isolated from an immunodeficient patient, a slight reactivity with OPV immune serum was observed, and no reactivity was observed between this virus and sIPV or Salk-IPV immune sera [10]. Whether this experimental result is due to altered vaccine antigenicity caused by the inactivation process of the Sabin virus or to the evolution of the OPV in humans, further investigation is needed from the perspective of polio vaccine research and disease control. Clearly, the type II iVDPV strain described above is an exceptional example that deserves further study. Therefore, this study investigated whether this iVDPV strain may be a representative node of poliovirus evolution in vaccine-immunized populations or a phenomenon that will pose new challenges for the practical application of polio vaccines.

## 2. Materials and Methods

### 2.1. Cells, Viruses, and Human Sera

Human embryonic kidney cells (HEK293 cells) and human rhabdomyosarcoma cells (RD cells) were grown as monolayers in Dulbecco’s modified Eagle medium (DMEM) supplemented with 10% fetal calf serum (FCS). A PV pseudovirus neutralization test (pPNT) was performed on RD cells [11]. PV pseudoviruses were produced in HEK293 cells. PV pseudoviruses containing the capsid protein from PV2 were created. The Institute of Medical Biology produced rabbit anti-IPV2 antibodies. Human sera were collected from healthy volunteers (aged 3 to 60 years) under informed consent, either from themselves or from a parent or legal guardian for minors. The experiments performed here were approved by the Committee for Ethical Regulation at the Ethics Committee of Guangxi CDC, China.

### 2.2. Construction of Poliovirus Capsid Expression Plasmids

Using vector pcDNA-P1-S2 as the template, the PV2 capsid protein-coding region was amplified via PCR using primers F2 (Sabin) and R2 (vector).

Using plasmid pKS435-EGFP-PV CAPSID as the template, the EGFP coding area was amplified via PCR using primers F1 (vector) and R1 (GFP). The primers are listed in Table S1 in the Supplementary Materials. To finish building the poliovirus capsid Sabin II expression plasmids, PKS435-EGFP-SabinII, homologous recombination took place at the ends of two DNA pieces under the influence of homologous recombinase.

Using the primer pairs R141S delF/A679D-R or A679D-F/RR141S delR, which are listed in Table S1 in the Supplementary Materials, PCR-based mutagenesis was used to construct 11 expression vectors of PV2 capsid protein variants with amino acid substitutions at positions 141, 679, or 680 in the backbone vector pKS435-EGFP-Sabin.

The plasmids containing the mutated sequences were subjected to sequence analysis to exclude the presence of unintended mutations.

### 2.3. T7-Transcribed Pseudovirus Backbone RNA In Vitro

RNA transcripts of PV replicons were obtained using a RiboMAX large-scale RNA production system and the T7 kit (Promega, Madison, WI, USA, P1320) with DraI-linearized DNA of pPV-Fluc mc, which encodes a PV replicon based on PV1, which has the firefly luciferase gene instead of the capsid-coding region as the template. The RNA was purified using phenol extraction [12,13].

### 2.4. Preparation of Sabin II and Mutation Sabin II Pseudovirus

PV pseudoviruses were developed using a previously published method with slight modifications [12]. Briefly, using Lipofectamine 2000 reagent (Invitrogen, Carlsbad, CA, USA, 11668019), confluent (30%) HEK293 cells in six-well plates were transfected with 3 µg of corresponding Sabin II and mutation Sabin II capsid expression vectors per well. The cells were then incubated for 24 h at 37 °C in 2 mL DMEM supplemented with 10% FCS per well. Using Lipofectamine 3000 reagent (Invitrogen, Carlsbad, CA, USA, L3000015), RNA transcripts were transfected into HEK293 cells transiently producing PV capsid proteins at 24 h post-infection (p.i.). After being harvested 24 h after the RNA transcripts were detected, the cells were kept at −80 °C. By counting the number of RD cells infected with PV pseudoviruses eight hours after infection, the infectious unit of the PV pseudovirus stock solution was ascertained. Luciferase activity was determined 8 h after the virus and cells were inserted into 384-well plates [14].

### 2.5. Pseudovirus-Based Neutralization Assay

pPNT was performed as reported previously with modifications [15] using 384-well plates for the neutralization assay. After serum inactivation, 2-fold serially diluted sera samples from 1/4 to 1/4096 dilution were prepared and then inoculated with an equal volume of PV pseudovirus solution containing 400 infectious units [IU] for type 2 at a final volume of 10 µL per well at 37 °C for 3 h. The RD cells were placed into wells at 20 μL/well for a total of 1 × 10^6^/mL. The cells were incubated for 8 h in a 37 °C incubator, and luciferase activity was determined using a luciferase assay kit (Promega, Madison, WI, USA, E1500). The luciferase activity of the infected cells was measured at 8 h post-infection with the Luciferase Assay System (ThermoFisher, Waltham, MA, USA, model 3001) according to the manufacturer’s instructions. Pseudovirus infection was calculated as a percentage of the luciferase activity of the infected cells, where the luciferase activity in mock-treated cells was taken as 100% (standard deviations, about 20% of the means). The neutralizing antibody titers of the serum against type 2 PV were determined as the highest dilution of the serum that inhibited two types of Sabin PV pseudovirus infection at 5% [13,14,15,16].

### 2.6. Bioinformation Search Method

The iVDPV2 nucleotide sequences of the complete genome and capsid region were searched in GenBank using the keywords “(immunodeficient or immunodeficiency) and poliovirus 2”. As of 25 March 2022, there were 33 type 2 entries out of the initial 402 entries searched. Reference sequences, partial sequences, repetitive sequences, and Sabin-like sequences (<6 nt differences) were removed. Following a detailed literature review, four additional sequences not identified in the initial GenBank search were added.

### 2.7. Establishment of Homology Models

The three-dimensional structural models of four coat proteins, VP1, VP2, VP3, and VP4, of iVDPV were predicted and analyzed using SWISS-MODEL online analysis software based on a homology algorithm [17,18]. The PDB database Poliovirus Type II (strain Lansing) (PDB sequence number: 1EAH) was used as a template to predict the iVDPV and Sabin II conformations in SWISS-MODEL, respectively. The predicted conformations were simultaneously displayed using Pymol 2.1 software/SPDBV 4 software [19]. The predicted iVDPV and Sabin II conformations were fitted to find the amino acid positions with spatial conformational differences in the antigenic epitopes [20].

### 2.8. Nucleotide Sequence Registration Numbers

The complete genome sequences of the five iVDPV2 strains described in this study are registered under the following registration numbers: GU390707, DQ890387, FJ517648, AJ544513, and AY177685. The registration numbers for the phylogenetic analyses reported herein from capsid sequences are iVDPV:KR817050–KR817060; GU390704–GU390706; cVDPV:MG212487; KJ170558; JX275352; KJ170561; DQ890385; KJ170563; KU598886; and HM107835; and the wild virus strains are KR817062 and V01149.

### 2.9. Phylogenetic Tree Analysis

Differences in the similarity ratio of multiple sequences to the primary structure were calculated using the Clusalw [21] and Muscle [22] methods of the Mega-x program, and phylogenetic tree maps were constructed using the maximum likelihood method [23]. Representative related sequences were included in the phylogenetic analysis. Related sequences were selected through comparisons with the parental Sabin II strain (accession no. AY184220) to obtain the total number of sequence substitutions, with VP1 > 0.6% being set for related sequences based on the percentage of amino acids substituted. Variant sites of iVDPV strains, poliovirus Sabin II, and MEF-1 strains were identified by comparing the amino acid sequences in the neutralization site region. Four important immune epitopes are known, which we refer to as NAg sites (NAg I, NAg II, NAg IIIa, and NAg IIIb) [24,25].

### 2.10. Statistical Analysis

A Kruskal–Wallis test and one-way ANOVA were performed using SPSS 16.0 analysis software to compare the cross-neutralizing antibody titers for each mutated pseudovirus strain.

## 3. Results

### 3.1. Neutralization Site Analysis

The antigenic epitope sequence of the patient-isolated virus iVDPV (GU390707) was compared with the reference sequence Sabin II virus, the wild strain (MEF-1), and strain p712 (Table 1). The amino acid side chains were classified as nonpolar, polar, positively charged, and negatively charged. As shown in Table 1, 16 amino acid mutations were identified in the antigenic epitopes. Of these, 14 underwent changes in charged properties, specifically, R 141 S (positive–polar), T 233 K (polar–positive), A235T (nonpolar–polar), T236D (polar–negative), and N 241 K (polar–positive) for VP2; T415A (polar–nonpolar) and H417N (positive–polar) for VP3; and S418L (polar–nonpolar), T420E (polar–negative), K677E (positive–negative), A679 defective (nonpolar), S680D (polar–negative), T801A (polar–nonpolar), and G867E (nonpolar–negative) for VP1. In addition, because there is a deletion at one amino acid position in 679 and 680 of VP1 and it is not possible to determine which position it is, there is an additional case of A 679 D and S 680 deletion (negative polarity) in 679 and 680 of VP1. In addition, two strains of iVDPV (AY177685 and FJ517648) had surface-neutralizing epitopes that remained unchanged, but neurotoxicity was restored. The Ka/Ks were 0.23 at both coat protein P1 and antigenic epitopes.

### 3.2. Prediction of the 3D Spatial Conformation of the Capsid Protein of the iVDPV Isolate (GU390707) and Sabin Strain

We used the crystal structure of poliovirus type II (Lansing strain) to create homology models of the iVDPV type II variant (GU390707) (http://swissmodel.expasy.org/, accessed on 2 May 2020), which were visualized using Swiss-PDP viewer v4.0 software. Pairwise model comparisons of the Lansing strain and the iVDPV type II variant were performed via superimposition.

The model predictions of the substitution at two adjacent residue sites (679 and 680) may influence the B-C loop by shifting from a rigid alpha helix to a flexible loop structure. There are also two mutations from arginine (R) to serine (S) at position 141 in VP2 and from glycine (G) to glutamate (E) at position 866 in VP1, both of which exhibit few changes in spatial structure and are unchanged in the loop configuration but do not overlap completely. By examining their interactions, it was found that the mutation of residue 141 of VP2 was more variable, while the mutation of VP1 at position 866 overlapped with the Sabin strain at that site. Therefore, we selected residues 679 and 680 of VP1 and 141 of VP2 for the subsequent point mutation study.

### 3.3. Mutant Sabin II Pseudoviruses Against Rabbit Polyclonal Antibody and Human Serum

**Figure 1:**
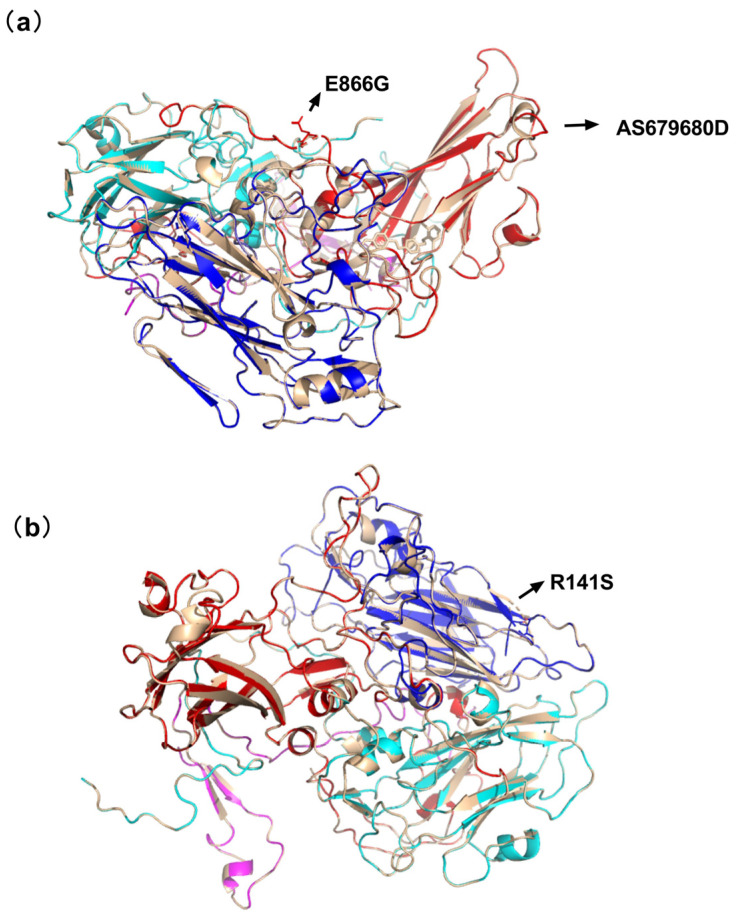
Homology modeling of neutralization epitope variation of type II Sabin poliovirus. The homology model of type II Sabin poliovirus was based on poliovirus type II (strain Lansing) (protein database [PDB] key number: 1eah). VP1 was red, VP2 was blue, VP3 was cyan, VP4 was pink, and VP1–VP4 of the Sabin strain was gray. (**a**) neutralization epitope variation of VP1 of type II Sabin poliovirus. (**b**) neutralization epitope variation of VP2 of type II Sabin poliovirus.

We next constructed 11 mutant Sabin II PsVs, Sabin II PsVs, and iVDPV2 PsVs (GU390707) (141S,679del, 679D,680D,680del, 141 680Sdel, 141 679SD, 141S, 141 679 S del, 141 680 SD, 679 680 Sdel, 141 679 680 SDdel) to examine the potential impact of this BC loop residue on sensitivity to cross-neutralizing antibodies. As shown in Figure 1, after mutating three amino acids at the NAg site of the Sabin II strain, we found that these mutant pseudoviruses decreased the sensitivity of cross-neutralizing antibodies compared with the Sabin strain. The source of the antibodies appeared to have an impact on the degree of these sensitivity differences for some variants. In terms of rabbit antibody sensitivity, variation 141 679del was 0.023 times more sensitive than Sabin II PsVs, whereas variant iVDPV was 0.125 times more sensitive. In the cross-neutralization test with rabbit polyclonal antibody, the neutralization sensitivity of the other ten strains was significantly different from that of iVDPV (*p* > 0.05). In the human serum, the cross-neutralization test was slightly different from that of the rabbit polyclonal antibody. In terms of human serum sensitivity, variation 141 679del was 0.13 times more sensitive than Sabin II PsVs, whereas variant iVDPV was 0.27 times more sensitive. All 10 mutants, except for the 141 680D mutant strain, showed an increase in neutralizing antibody titers against three human sera compared with the iVDPV strain (*p* < 0.05). In summary, for the two amino acids 679 and 680 with an AS mutation to D, it can be determined that a deletion occurred at amino acid position 679, while amino acid position 680 had a serine mutation to aspartate (D).

## 4. Discussion

In the 1990s, nearly 350,000 children were infected with paralytic polio each year. A worldwide IPV and OPV vaccination effort brought wild-type poliovirus to the brink of global eradication using this effective vaccine [25]. However, the global eradication of poliovirus is hampered by epidemics of vaccine-derived poliovirus. In 2024, nearly 7 years after the global shift to bivalent oral poliovirus vaccine (bOPV), the world now faces increasing cVDPV2 outbreaks in parts of Southeast Asia and the Middle East [26]. From January 2023 to June 2024, 532 AFPs of cVDPV2 cases were confirmed in 26 countries [27]. The number and extent of these outbreaks have exceeded expectations, and cVDPV2 outbreaks have become a major challenge in the final stages of eradication. Of the 113 patients with poliovirus-secreting primary immunodeficiencies (PIDs) identified between 1962 and 2016, 69.08% secreted type II PV [28]. Meanwhile, we learned that the immune serum pairs of sIPV and wIPV are poorly neutralized or not neutralized at all with some iVDPV strains (in this study, type II iVDPV, GU390707) [10]. Although interrupting the OPV can stop the emergence of cVDPV, dealing with the threat posed by iVDPV is not straightforward; the prevalence of chronic excretions in the population is currently unknown, and identifying and monitoring VDPV requires a better understanding of the biology of chronic infections and a significant amount of surveillance work [29].

A total of 740 nucleotide differences were discovered between GU390707 and the type II Sabin strain, giving rise to 84 amino acid differences. A comparison between the P1 protein of the iVDPV strain (GU390707) and the Sabin strain revealed 16 amino acid differences in the major neutralizing antigenic epitopes (NAG) on VP1, VP2, and VP3, of which 14 amino acids changed in polarity or charge. To predict the surface structure of the viral particles and the spatial conformational changes in the coat proteins of Sabin II and iVDPV2 strains, 3D structural simulations in SWISS-MODEL online software were performed, revealing that changes in three amino acid residues affected the spatial conformation of the viruses in NAG. Among the predicted results of the 3D structure, only the simultaneous mutation of the two sites 679 and 680 made the 3D structure change more when the rigid structure α-helix was changed to a loop structure, which increased the flexibility of the BC loop. The BC loop is located at the tip of each of the fivefold axes of the stellate tableau. It does not have a fixed conformation, but under appropriate conditions, such as temperature change, it can be very flexible to adjust the structure [30]. This shows that the antigenicity of PV is closely related to the spatial conformation. NAg site IIa has two amino acid changes (VP1, S800D, and T801A), in which amino acid 801 changes from a polar amino acid to a nonpolar amino acid, and 800 and 801 are located in the GH ring. NAg site IIb has three amino acids at the very tip of the EF ring in VP2 (T233K, A235T, T236D, N241K). NAg site IIIb, R141S on VP3, which is shown by the force of action, reveals that the β-folding turns into a loop structure. NAg site IIIa exhibited changes in four amino acids on VP3 (T415A, H417N, S418L, and T420 E) and is located in the second β-fold [31,32]. This antigenic difference may be due to a large number of amino acid substitutions at neutralizing antigen (NAg) sites I, II, and IIIa aggregated near the surface or within the ring.

By simulating the spatial conformation, the proposed relevant antigenic epitope sites were selected and subjected to cross-neutralization protection assays to verify the effect of the simulated coat protein alterations on the neutralization epitope and the effect of these changes on the antisera neutralization capacity of the vaccine.

**Figure 2:**
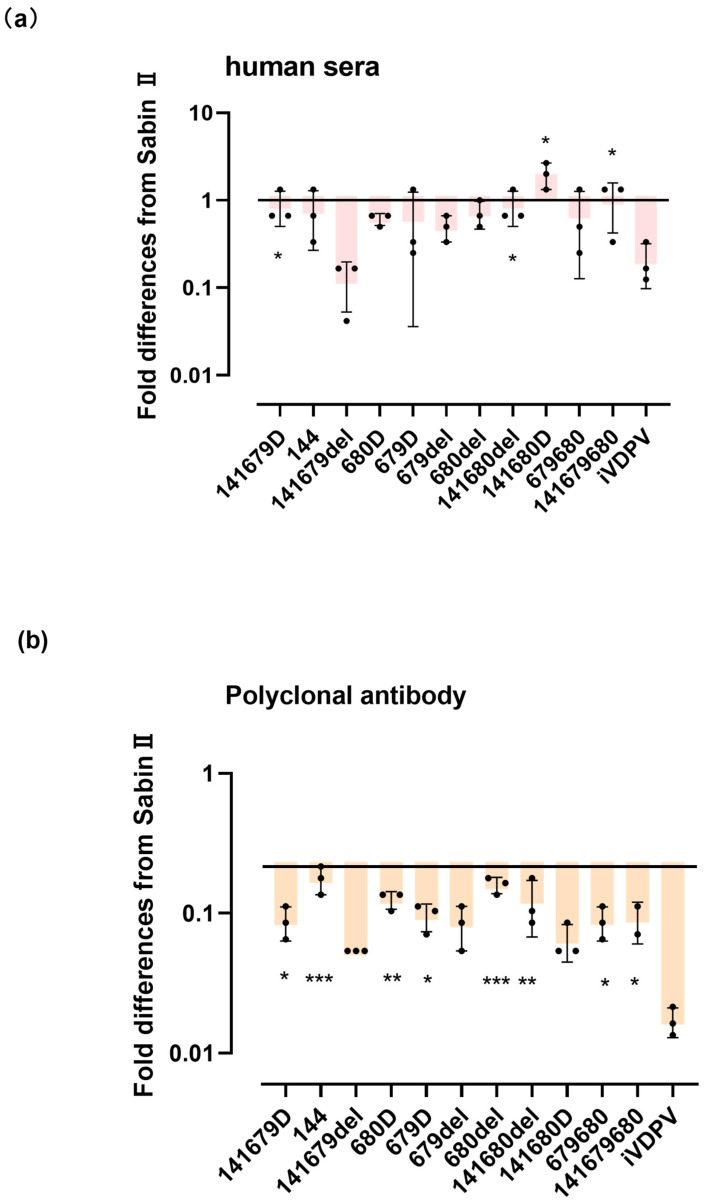
Neutralization sensitivities of mutant type II Sabin pseudovirus. Box (median, IQR) and whisker (10th and 90th percentiles) plots of fold differences in neutralization titer from that of the iVDPV pseudovirus for the type II Sabin pseudovirus and their indicated mutants in polyclonal antibody (*n* = 3) and human sera (*n* = 3). *, *p* < 0.05 **, *p* < 0.01 ***, *p* < 0.001 (Wilcoxon paired sign-rank test). (**a**) In human sera, the fold difference in neutralization titers between the type II Sabin pseudovirus and its mutants of pseudovirus. (**b**) In polyclonal antibody, the fold difference in neutralization titers between the type II Sabin pseudovirus and its mutants of pseudovirus.

Eleven mutant pseudoviruses of the three mutation combinations were tested for cross-neutralization against human sera and rabbit polyclonal antibodies. The results of the cross-neutralization tests showed that the protection afforded by the sera was largely reduced by the mutation of the neutralization site, with the greatest effect being the simultaneous mutation of the 141 site and deletion of the 679 site, followed by the simultaneous mutation of the 141 and 680 sites. The protection of serum and rabbit polyclonal antibodies was weakened by either single-point mutation or deletion at the 679, 680, and 141 sites of the P1 protein, but the protection of rabbit polyclonal antibodies decreased more significantly. However, the cross-neutralization tests with rabbit polyclonal antibodies and human sera showed that the attenuated protection of pseudoviruses with three site mutations was not the most significant (Figure 2). This may be due to the fact that the mutation of just the three sites together causes a change in the spatial conformation of the antigenic epitope, but there is no real positive correlation between the neutralizing epitope mutation and the reduced response to the antiserum. It is not the case that the more mutations there are, the weaker the antiserum response. The trivalent inactivated Sabin vaccine and monovalent type II Sabin produce different antibody pools in immunized animals or humans. In addition, the source of the antibody may influence the difference in sensitivity to neutralizing site variants. An alternative explanation for the mechanism of action of the 679 and 680 mutations is that it regulates the flexibility of the capsid by altering the conformation of the VP1 BC loop (Figure 2). The rearrangement of the VP1 BC loop may alter the structure of the neighboring DE and HI loops and create a new thermodynamic state on the fivefold axis of symmetry [33].

**Figure 3:**
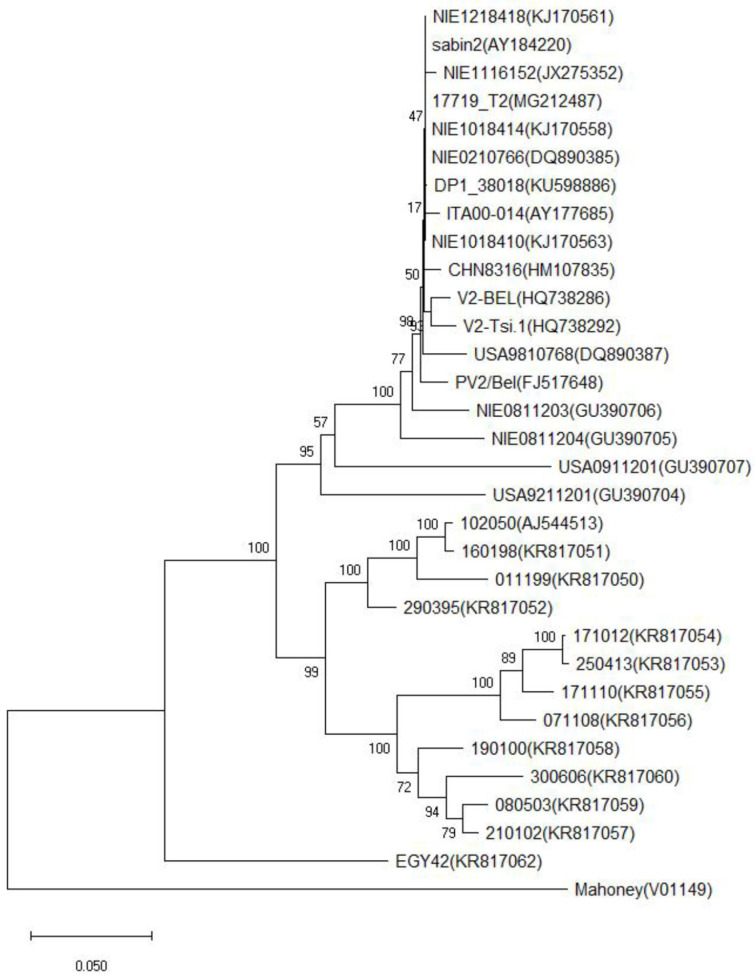
Neighbor-joining trees summarizing sequence relationships among the type 2 iVDPV isolates and VDPV2 strain across the P1/capsid region.

In order to elucidate the differentiation and evolution of type II iVDPV, the viral capsid protein P1 sequence was subjected to multiple sequence comparisons and phylogenetic analyses, which showed that type II iVDPV (GU390707) is classified into a separate taxon with a different pathway of differentiation from Sabin II and is distinct from the genetic taxa of other iVDPVs. There are five major branches in this evolutionary tree (Figure 3).

To determine the antigenic changes caused by the prolonged excretion of the virulent strains in humans, the three sites (amino acids 101 and 102 of VP1, amino acid 72 of VP2) where the Sabin strain and an iVDPV strain had mutations at the NAg site and a change in spatial conformation were selected (Figure 1). By merging the three mutant locations, eleven pseudovirus strains were created, including the type II Sabin strain and an iVDPV strain that underwent cross-neutralization testing against three human sera and one rabbit polyclonal antibody.

The cross-neutralization tests of the neutralization epitope mutant pseudoviruses using human and rabbit antibodies revealed that neutralization site mutation significantly reduced the protection offered by the sera. The largest effect was observed for the simultaneous mutation of the 141 site and deletion of the 679 site, followed by the simultaneous mutation of the 141 and 680 sites. The 679 single-point deletion significantly reduced the protective effect of the serum.

Cross-neutralization studies employing both rabbit antibody and human sera revealed that viruses with simultaneous mutations at all three RAS141679680S delD/Ddel sites responded with the rabbit antibody and human sera, and the effect of the sera on the protection of this strain was statistically significant (Figure 2). The monovalent type II Sabin vaccine and the trivalent inactivated Sabin immunization produce different antibody pools when given to humans or animals, respectively. Furthermore, the origin of the antibody may have affected the variance in sensitivity to neutralizing-site alterations.

Following the primary structure comparison and homology modeling of the iVDPV (GU390707) and Sabin strains, a protein tertiary structure analysis revealed spatial conformational alterations at amino acids 679 and 680 of VP1 and amino acid 141 of VP2. After creating eleven different combinations of three-point mutant pseudoviruses and testing their neutralization with immune serum, we discovered that immune serum would be the most effective in neutralizing pseudoviruses that were concurrently altered with the Sabin strain 141 and 679. After testing 11 pseudoviruses for affinity with the PV receptor, it was shown that mutations of VP1 at locations 679 and 680 could enhance the pseudovirus attraction, with position 679 showing the greatest and most significant increase.

## Figures and Tables

**Table 1:** Polio II virus neutralization antigen mutation analysis.

Type II	VP2			VP3		VP1		
	IIIb 140–142	II 232–241	II 336–338	IIIa 394–401	IIIb 410–420	I 666–684	IIa 800–804	IIIa 864–867
SabinII	WRK	DTNATNPARN	PRT	PLNLTSQR	VELSDTAHSDT	AIIEVDNDAPTKRASRLFS	STEGD	KDGLT
MEF-1	WRK	DTNATNPARN	PRT	PLNLTNQR	VELNDAAHSDT	AIIEVDNDAPTKRASKLFS	STEGD	KDGLA
P712	WRK	DTNATNPARN	PRT	PLNLTSQR	VELSDTAHSDT	AIIEVDNDAPTKRASRLFS	STEGD	KDGLT
cVDPV2	WRK	DTNATNPARN	PRT	PLNLTSQR	VELSDMARSDT	AIIEVDNDAPTKRASRLFS	STEGD	KDGLT
iVDPV	WSK	DKNTDNPARK	PRT	PLNLTSQR	VELSDAANLDE	AIIEVENDAPTERXDRLFS	QAEGD	KDELT

Red letters represents the mutated sites of the other strains compared to polio SabinII virus.

## Data Availability

Data are contained within the article and Supplementary Materials.

## Supplementary Materials

The following supporting information can be downloaded at: https://www.mdpi.com/article/10.3390/v16111761/s1, Table S1: Primer sequences required for plasmid construction.

## References

[1] Dawson L. (2004). The Salk Polio Vaccine Trial of 1954: Risks, randomization and public involvement in research. Clin. Trials.

[2] Poirier B., Morgeaux S., Fuchs F. (2002). The assessment of OPV vaccines by the monkey neurovirulence test: Why and how to qualify the experts in histology of central nervous system. Vaccine.

[3] McDonald S.L., Weldon W.C., Wei L., Chen Q., Shaw J., Zhao K., Jorba J., Kew O.M., Pallansch M.A., Burns C.C. (2020). Neutralization capacity of highly divergent type 2 vaccine-derived polioviruses from immunodeficient patients. Vaccine.

[4] Quarleri J. (2023). Poliomyelitis is a current challenge: Long-term sequelae and circulating vaccine-derived poliovirus. Geroscience.

[5] Anis E., Kopel E., Singer S.R., Kaliner E., Moerman L., Moran-Gilad J., Sofer D., Manor Y., Shulman L.M., Mendelson E. (2013). Insidious Reintroduction of Wild Poliovirus into Israel, 2013. Eurosurveillance.

[6] Minor P.D. (2016). An Introduction to Poliovirus: Pathogenesis, Vaccination, and the Endgame for Global Eradication. Methods Mol. Biol..

[7] Aylward B., Yamada T. (2011). The polio endgame. N. Engl. J. Med..

[8] Nathanson N. (2011). Eradication of poliovirus: Fighting fire with fire. J. Infect. Dis..

[9] Bhaumik S. (2012). Polio eradication: Current status and challenges. J. Family Med. Prim. Care.

[10] Sun M., Li C., Xu W., Liao G., Li R., Zhou J., Li Y., Cai W., Yan D., Che Y. (2017). Immune Serum from Sabin Inactivated Poliovirus Vaccine Immunization Neutralizes Multiple Individual Wild and Vaccine-Derived Polioviruses. Clin. Infect. Dis..

[11] Arita M., Iwai M., Wakita T., Shimizu H. (2011). Development of a poliovirus neutralization test with poliovirus pseudovirus for measurement of neutralizing antibody titer in human serum. Clin. Vaccine Immunol..

[12] Arita M., Nagata N., Sata T., Miyamura T., Shimizu H. (2006). Quantitative analysis of poliomyelitis-like paralysis in mice induced by a poliovirus replicon. J. Gen. Virol..

[13] Viktorova E.G., Khattar S., Samal S., Belov G.A. (2018). Poliovirus Replicon RNA Generation, Transfection, Packaging, and Quantitation of Replication. Curr. Protoc. Microbiol..

[14] Arita M., Iwai-Itamochi M. (2019). Evaluation of antigenic differences between wild and Sabin vaccine strains of poliovirus using the pseudovirus neutralization test. Sci. Rep..

[15] Zhang H., An D., Liu W., Mao Q., Jin J., Xu L., Sun S., Jiang L., Li X., Shao J. (2014). Analysis of cross-reactive neutralizing antibodies in human HFMD serum with an EV71 pseudovirus-based assay. PLoS ONE..

[16] Liu S., Song D., Bai H., Lu W., Dai X., Hao C., Zhang Z., Guo H., Zhang Y., Li X. (2017). A safe and reliable neutralization assay based on pseudovirus to measure neutralizing antibody titer against poliovirus. J. Med. Virol..

[17] Arnold K., Bordoli L., Kopp J., Schwede T. (2006). The SWISS-MODEL workspace: A web-based environment for protein structure homology modelling. Bioinformatics.

[18] Kopp J., Schwede T. (2004). The SWISS-MODEL Repository of annotated three-dimensional protein structure homology models. Nucleic Acids Res..

[19] Rigsby R.E., Parker A.B. (2016). Using the PyMOL Application to Reinforce Visual Understanding of Protein Structure. Biochem. Mol. Biol. Educ..

[20] Patel V., Ferguson M., Minor P.D. (1993). Antigenic sites on type 2 poliovirus. Virology.

[21] Larkin M.A., Blackshields G., Brown N.P., Chenna R., McGettigan P.A., McWilliam H., Valentin F., Wallace I.M., Wilm A., Lopez R. (2007). Clustal W and Clustal X version 2.0. Bioinformatics.

[22] Edgar R.C. (2004). MUSCLE: Multiple sequence alignment with high accuracy and high throughput. Nucleic Acids Res..

[23] Kimura M. (1980). A simple method for estimating evolutionary rate of base substitutions through comparative studies of nucleotide sequences. J. Mol. Evol..

[24] Minor P.D. (1990). Antigenic structure of picornaviruses. Curr. Top. Microbiol. Immunol..

[25] Bakker W.A., Thomassen Y.E., van’t Oever A.G., Westdijk J., van Oijen M.G., Sundermann L.C., van’t Veld P., Sleeman E., van Nimwegen F.W., Hamidi A. (2011). Inactivated polio vaccine development for technology transfer using attenuated Sabin poliovirus strains to shift from Salk-IPV to Sabin-IPV. Vaccine.

[26] Bigouette J.P., Henderson E., Traoré M.A., Wassilak S.G.F., Jorba J., Mahoney F., Bolu O., Diop O.M., Burns C.C. (2023). Update on Vaccine-Derived Poliovirus Outbreaks—Worldwide, January 2021–December 2022. MMWR Morb. Mortal. Wkly. Rep..

[27] Namageyo-Funa A., Greene S.A., Henderson E., Traoré M.A., Shaukat S., Bigouette J.P., Jorba J., Wiesen E., Bolu O., Diop O.M. (2024). Update on Vaccine-Derived Poliovirus Outbreaks—Worldwide, January 2023–June 2024. MMWR Morb. Mortal. Wkly. Rep..

[28] Shaghaghi M., Soleyman-Jahi S., Abolhassani H., Yazdani R., Azizi G., Rezaei N., Barbouche M.R., McKinlay M.A., Aghamohammadi A. (2018). New insights into physiopathology of immunodeficiency-associated vaccine-derived poliovirus infection; systematic review of over 5 decades of data. Vaccine.

[29] Howard W., Moonsamy S., Seakamela L., Jallow S., Modiko F., du Plessis H., Sibiya R., Kamupira M., Maseti E., Suchard M. (2021). Sensitivity of the acute flaccid paralysis surveillance system for poliovirus in South Africa, 2016–2019. J. Med. Microbiol..

[30] Lin J., Cheng N., Hogle J.M., Steven A.C., Belnap D.M. (2013). Conformational Shift of a Major Poliovirus Antigen Confirmed by Immuno-Cryogenic Electron Microscopy. J. Immunol..

[31] Blomqvist S., Bruu A.L., Stenvik M., Hovi T. (2003). Characterization of a recombinant type 3/type 2 poliovirus isolated from a healthy vaccinee and containing a chimeric capsid protein VP1. J. Gen. Virol..

[32] Page G.S., Mosser A.G., Hogle J.M., Filman D.J., Rueckert R.R., Chow M. (1988). Three-dimensional structure of poliovirus serotype 1 neutralizing determinants. J. Virol..

[33] Lin Y., Racaniello V.R. (2017). Polioviruses that bind a chimeric Pvr–nectin-2 protein identify capsid residues involved in receptor interaction. Virology.

