# Supplementary material for: Antisera-Neutralizing Capacity of a Highly Evolved Type 2 Vaccine-Derived Poliovirus from an Immunodeficient Patient
Source: Viruses. 2024 Nov 12;16(11):1761. doi: 10.3390/v16111761 (PMC11598955; doi:10.3390/v16111761)
Supplement: Supplementary file 1 [file viruses-16-01761-s001.zip › viruses-3245760-supplementary.pdf]

Table S1. The accession numbers for PV

Accession numbers	Country	Type
AY238473	USA	PV2(MEF-1)
X00595	USA	PV2(p712)
GU390707	USA	iVDPV2
DQ890387	Nigerian	iVDPV2
FJ517648	Belarus	iVDPV2
AJ544513	UK	iVDPV2
AY177685	Italy	iVDPV2
KR817050	UK	iVDPV2
KR817051	UK	iVDPV2
KR817052	UK	iVDPV2
KR817053	UK	iVDPV2
KR817054	UK	iVDPV2
KR817055	UK	iVDPV2
KR817056	UK	iVDPV2
KR817057	UK	iVDPV2
KR817058	UK	iVDPV2
KR817059	UK	iVDPV2
KR817060	UK	iVDPV2
GU390704	USA	iVDPV2
GU390705	USA	iVDPV2
GU390706	USA	iVDPV2
MG212487	Russia	cVDPV2
KJ170558	Nigerian	cVDPV2
JX275352	Nigerian	cVDPV2
KJ170561	Nigerian	cVDPV2
DQ890385	Nigerian	cVDPV2
KJ170563	Nigerian	cVDPV2
KU598886	Russia	cVDPV2
HM107835	China	cVDPV2
KR817062	Egypt	PV2(EGY42)
V01149	USA	PV1 (Mahoney)
